# Partial Least Squares Structural Equation Path Modelling Determined Predictors of Students Reported Human Cadaver Dissection Activity

**DOI:** 10.4236/fmar.2020.82003

**Published:** 2020-03-17

**Authors:** Ian G. Munabi, William Buwembo

**Affiliations:** Department of Anatomy, College of Health Sciences, Makerere University, Kampala, Uganda

**Keywords:** Anatomy, Dissection, Cadaver, Partial Least Squares Structural Equation Modeling

## Abstract

Human cadaver dissection remains a core and preferred method of anatomical instruction at most low- and middle-income health professional training institutions. Dissection, which is both traumatic and stressful, sets the tone of the students’ responses to later and or similar stressful learning opportunities like the post-mortems or care for terminally ill patients. Partial least squares structural equation modelling was used to determine the effect of the students’: personality, perception of the learning environment, learning approach, and effect of the environment on the student, on undergraduate health professional student’s activity in the human cadaver dissection room. This was a secondary analysis of previously collected data from a cross sectional survey of undergraduate health professional students. We found that personality type and perception of the environment had a positive effect on dissection room activity. Approach to learning and being affected by the dissection room experience (impact), had a negative effect on dissection room activity. All the above effects on dissection room activity were not significant. This study showed that personality, perception of the learning environment, learning approach and effect of the environment on the student, had effects on undergraduate health professional student’s activity in the human cadaver dissection room. The modelled effects are opportunities for educational interventions aimed at increasing student activity in the dissection room.

## 1. Introduction

All over the world changes in medical education have resulted in greater focus on what and how students learn [1] [2] [3]. This is most clearly shown in the global move towards the adoption of various forms of student-centered approaches to learning, of which, Problem Based Learning (PBL) is an example [4] [5] [6]. The student-centered human cadaver gross anatomy dissection room learning experience at Makerere University is organized around, small groups working as teams to cover material on various aspects of studying the human body [7]. Cadaver dissection is considered a key aspect of learning of human anatomy at Makerere University and other medical schools around the world [8]. Through gross anatomy health professional students quickly get exposed to the: medical language and culture, ethics, teamwork, and various professional behaviors in addition to developing a three-dimensional orientation of the human body as they work in teams around a preserved human body following instructions provided in a dissection manual. In high resource settings other methods of learning anatomy include the use of pre dissected specimens, plastination and computer aids [9]. While there exist several arguments for and against use of the human body as a tool for teaching anatomy [10], it remains the preferred method of anatomical instruction in most low resource settings on the African continent [2] [7] [11] [12].

The educational content and the context in which the learning occurs can affect the quality of learning [3] [13]. In the case of Anatomy cadaver-based dissection, the content is what must be learned from the cadaver and the context is the dissection laboratory. To some students the cadaver may be viewed as a source of negative or noxious stimuli that can affect the students’ progress through the curriculums intended learning pathway. Fortunately, most students are able to quickly adjust and proceed to learn effectively [14]. In more developed settings, about 2% - 5% of the students in each class will find the cadaver room experience persistently stressful enough to affect their studies [15]. Similarly, in Saudi Arabia, and Jordan [16] [17], 30% of the health professional students surveyed found the cadaver room experience persistently stressful enough to affect their studies. Conversely, it has been argued that the use of the human cadaver dissection helps health professional students to learn how to control their emotions [14]. Other authors argue that human cadaver dissection is a traumatic and stress full exercise for students that sets the tone for their responses to later similarly stressful learning opportunities like the post-mortems or care for terminally ill patients [18] [19] [20].

Health professional students who miss the available scheduled learning opportunities as prescribed by the curricula they follow may later become a danger to either themselves and or their patients [1] [21]. From the institutional perspective these students if not identified early have the potential to affect the institutions’ reputation or brand in the marketplace [1] [21]. This makes it important to put in place mechanisms or measures for early prediction and or identification of students in need of early remedial action. This is especially important in low resource settings where due to shortages in the human resource for health, the public service system, usually deploys recently qualified health professionals with bachelor’s degree training as its frontline health workers. In the current study four validated questionnaires were used as measures for the latent variables corresponding to: personality [22], perception of the learning environment [23], effect of the environment on the student [24] [24], and the student’s approach to learning [22]. It was hypothesized that the interactions between the latent variables could fit into a structural equation model predicting student’s activity in the dissection room.

Partial least squares structural equation modelling (PLS-SEM), is the preferred modelling method for predictive and or exploratory studies [26]. PLS-SEM is also preferred where sample sizes are small and or with missing values as commonly encountered in secondary analyses [26]. The methods for implementing PLS-SEM that were not available at the time of the study are now widely available and implemented as free open source packages that include: semPLS [27], PLS-PM [28] and MatrixPLS [29] in the R statistical computing software environment [30]. The availability of the PLS-SEM packages and increased computing power for ordinary PC users made it possible to do the secondary analysis in this study [26]. For this secondary analysis, partial least squares SEM was used to determine the effect of the students’: personality [22], perception of the learning environment [23], learning approach [22] and effect of the environment on the student [24] [25], on undergraduate health professional student’s activity in the human cadaver dissection room.

## 2. Methods

The data for this secondary analysis was collected as part of a dissertation research project for the award of a Master’s in Health Professional Education. The work in this dissertation was done at the Anatomy Department of the then Faculty of Medicine at Makerere University. In 2008, at the time of data collection the Makerere University Faculty of Medicine was in its fourth year of rolling out an innovative student-centered PBL curriculum [31] [32]. The Anatomy Department, one of the oldest departments in the medical school, has two cadaver dissection rooms with 22 stainless steel dissection tables. Two tables are reserved for the post graduate students in the smaller graduate dissection laboratory. The remaining 20 cadaver tables serve the undergraduate class in the larger undergraduate’s students dissection laboratory. Each table has a formalin/phenol solution preserved human body that is used over the course of one year for student dissection. This student dissection is done with the support of faculty following the instructions in the Cunningham’s Dissection Manual volumes One, Two and Three.

The study participants for this study were the 2008/2009 academic year, first- and second-year undergraduate health professional students participating in anatomy dissection programs of the department. These students were on the following academic programs: Bachelor of Medicine and Bachelor of Surgery, Bachelor of Nursing, Bachelor of Medical Radiology, Bachelor of Dentistry and Bachelor of Pharmacy. The average number of students in the dissection room at the time was 155 undergraduate students at any one time. Most of the students were on average between 18 - 19 years of age by the time they started their respective programs. The data base from the primary study had a total of 96 consecutively sampled respondents. The demographic characteristics of the 96 respondents are summarized in Table 1. For a model with five (5) latent variables and 22 measured variables with 0.05 level of significance, needed a sample of minimum samples size of 77 participants for a moderate effect size of 0.4 that was calculated using an online sample size calculator for structural equation models [33]. The previously collected sample of 96 participants records were more than adequate.

**Figure 1.**
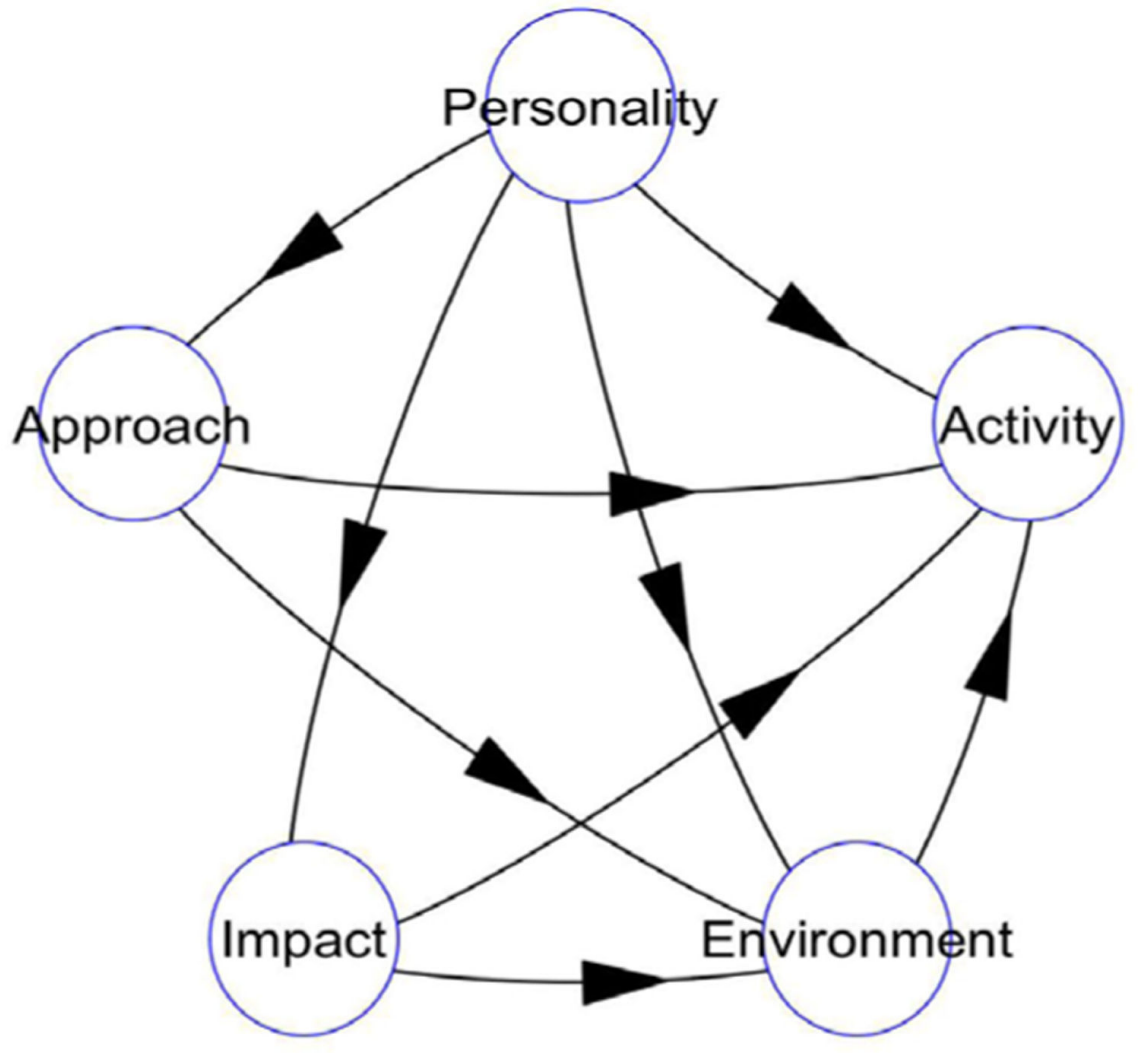
Structural equation model. Figure summary: figure 1 provides a visual representation of the final structural equation model in which the various relationships between the constructs measured by the different tools, and their corresponding latent variables (personality, approach to learning (Approach), impact of the dissection experience (Impact) and perception of the environment (Environment)), that all predict the learner’s activity (Activity), in the dissection room.

As shown in Table 2, the original study made use of four validated questionnaires that are described in more details as follows: 1) For personality, the revised Eysenck Personality Questionnaire (EPQ) [22], was used. This questionnaire has a set of twenty items that are easy and quick to answer. The information generated from the questionnaire is analyzed to give a score for two domains: neuroticism and introvert/extrovert [22]. The tool has been used for evaluating personalities of students doing anatomy in other settings [22]. 2) For the approaches the revised Biggs study questionnaire was used to assess the students study approaches [35]. The tool has already been used on undergraduate students studying anatomy at another university [36]. It gives an indication of the respondents’ current approach to studying that will be either deep or superficial. For this study the scores from the tool were summed up to give four scores corresponding to deep methods, deep strategies, superficial methods and superficial strategies. 3) For impact the Impact of Events Scale-revised (IES-R) tool [24], is made up of 22 questions that map onto 3 subscales (introversion, avoidance and hyper arousal), and describe the effect of a traumatic event or situation on the individual. The IES-R is a recognized tool for identifying individuals with mild forms of the post-traumatic stress disorder [37] [38] [39]. This is especially good for measuring the effect of cadaver dissection on the student [24]. 4) For the environment the Dundee Ready Education Environment Measure (DREEM) tool that has been used and validated in several communities all around the world [23], was used. It provides a measure of the student’s perception of the learning environment using five measures: perceptions of learning, perceptions of course organizers, academic self-perceptions, perceptions of atmosphere, and social self-perception. As shown in Figure 1, each of the domains and or summed up scores from the tools was used as the measured variable for latent variables in the structural equation model. All the tools used were validated, in multiple environments yielding consistent values for Cronbach’s Alpha greater than the minimum value of 0.7. To this combination of tools was included an additional set of questions to capture information about the students’ first encounter with cadavers, customs related to the dead, demographic information and students’ role in the dissection activities.

The final five-page questionnaire was tested on two students from each of the two participating student cohorts then distributed using student representatives in one sitting. The completed questionnaires from the self-selected participants were digitized using EPI6O4 for double data entry and validation, then exported as Dbase-4 files to R statistical computing software environment [30] for preliminary analysis using previously published keys for the different tools as shown in Table 2. The preliminary analysis generated a set of descriptive statistics corresponding to each of the tools used in the parent study. This preliminary analysis was followed by a partial least squares structural equation modelling using the PLS-PM package [28] to generate metrics of a model predicting students’ activity in the dissection room. The measured variables were modelled using mode A (reflective) to each latent variable shown in Figure 1. Because Partial least squares path modelling does not follow any distribution the identification significant paths and effects was based on the bootstrapping output after 15,000 boot replications [40]. The missing values were replaced using multiple imputation with the mice package [41], that was done using: predictive mean matching for numeric variables, logistic regression for categorical variables and multinomial logistic regression for ordered variables [42]. After imputation observations with more than 4 missing observations were dropped from further analysis. The level of significance 0.05 was used for all the calculations in this analysis. The quality control measures, and results of the exploratory structural equation modelling are presented following the Hair *et al.* (2019), reporting guidelines and other recommendations for PLS-PM [43] [44].

The student class representatives for the first- and second-year students at the time of the primary study were recruited as research assistants to distribute and later receive the duly filled questionnaires from only those students who agreed to participate in the study. These students, who voluntarily returned the study questionnaires, were assumed to have given their consent to participate after receiving a verbal explanation on the importance of participating in the study from their peer leaders. Students who choose not to return the questionnaires or even participate in the study continued to receive all the departmental services as they were entitled. No personal identifier marks were used on the questionnaire tools, during analysis or in the final report of the primary study. The university that was responsible for the award of the master’s degree classified education research as low risk research that did not need ethical approval. At the time of collecting data the study was in addition classified as part of the Anatomy Departments’ operational research thus not needing ethical review.

## 3. Results

There were 96 records from the respondents in the original study of which, 55/96 (57.29%) were complete. The remaining 41/96 (42.7%) records had a total of 70 missing observations distributed among the study variables as summarized in Table 3. Four records (4/96, 4.1%) with more than 4 missing values each were dropped. After imputation only 1/92 (1.1%) of the remaining records had one missing observation in three (3) variables. A generalized linear regression using the respondent’s year of study as the dependent variable did not demonstrate large differences in the regression coefficients of the variables with missing values compared with the same variables after imputation. In Table 3, note that the original model was better, as shown by its smaller Akaike information criterion (AIC) and larger pseudo R-squared (McFadden, Cox. and. Snell and Nagelkerke) values. Also, with the exception of age, the addition of the other variables in the database did not result into significant change in the model’s residual values. Age also had the largest number of missing values.

Table 4 provides a summary of the observations from the study questionnaires that includes information derived from the four study tools after imputation. In this study 36/93 (37.5%) of the respondents were female, 49/93 (52.7%) were first year undergraduate students and the average age for the group was 21.5 years old (SD 2.2). The majority of respondents (78/93, 83.9%) had seen a dead person, while 24/92 (26.1%) had touched a dead person prior to joining the medical school. Respondents who had seen a dead body were five time more likely to have also touched one compared to those that had never seen a dead body prior to joining the medical school. This was not significant (OR = 5.44, 95% CI 0.67 to 44.01). For the students’ preferred roles in human dead human body dissection: 40/93 (43.0%) respondents indicated they usually did the dissection, 21/93 (22.6%) read the dissection manual, 24/93 (25.8%) observed and asked questions but did not touch, 6/93 (6.5%) observed from a distance, and 2/93 (2.2%) indicated they never went to the dissection room. The first-year respondents were 0.38% more likely to be unemotionally affected by work on the human body compared to the second-year respondents (OR = 1.38, p-value = 0.46).

### 3.1. Observations from the Different Tools

#### 3.1.1. Dundee Ready Education Environment Measure (DREEM)

The mean score for the educational environment using the DREEM tool was 125.30 (SD 16.30). There were 4/93 (4.3%) students who thought there were many problems with the environment, 84/93 (90.3%) who thought there were more positives than negatives, and 5/93 (5.4%) who considered the environment was excellent. There was no significant difference in the means for the DREEM tool with respect to the year of study (t = −0.01, df = 90.9, p-value = 0.99), sex of the respondent (t = −0.80, df = 67.88, p-value = 0.43), or whether they reported having been affected by working on the human body (t = −0.20, df = 67.98, p-value = 0.84).

#### 3.1.2. Impact of the Events Scale-Revised (IES-R)

For the Impact of the events Scale-Revised (IES-R) tool, there were 38/93 (40.9%) students who were normal, 42/93 (45.1%) students who were moderately affected, and 13/93 (14%) who were severely affected by their most recent visit to the dissection room. The second-year students were twice as likely to be affected by the dissection room experience than the first-year students (odds ratio 1.91, 95% CI 0.52 - 2.75). Female students were 33% more likely to be affected by the dissection room experience than their male counterparts (Odds ratio 1.33, 95% CI 0.57 - 3.13). Student who had seen a dead human body prior to joining the medical school were more likely to be affected by the dissection room experience than those that had never seen a cadaver. This was significant (Odds ratio 6.93, 95% CI 1.97 - 32.58). Students who had touched a dead human body prior to joining the medical school were more likely to report being affected by the dissection room experience (odds ratio 1.24, 95% CI 0.48 - 3.32). Respondents who reported that the work in the dissection room had affected them emotionally, were three times more likely to be more affected by their most recent dissection room visit. This was significant (OR = 3.00, 95% CI 1.20 - 8.11).

#### 3.1.3. Study Approach

For the study approaches, there were 57/92 (62%) respondents using deep approaches to learning compared to 35/92 (38%) with more of the superficial approaches to learning. Respondents with a deep approach to learning had significantly higher scores for the environment than respondents with a superficial approach (t = −3.06, df = 83.09, p-value < 0.01). Respondents using deep approaches to learning were 0.41 times more likely to have adverse scores on the impact of dissection room event compared to those with superficial approaches (OR = 0.41, 95% CI 0.16 to 1.00). There was no significant difference in the odds of a respondent using either deep compared to superficial approaches of learning with respect to: Male sex (OR = 0.90, 95% CI 0.38 to 2.13), second Year of study (OR = 0.79, 95% CI 0.34 to 1.84), saw a dead human body before coming to medical school (OR = 1.24, 95% CI 0.38 to 3.93), touched a dead human body before coming to medical school (OR = 0.96, 95% CI 0.37 to 2.61), increasingly hands on role in dissection (OR = 1.11, 95% CI 0.76 to 1.62), and being emotionally affected by dissection (OR = 0.48, 95% CI 0.19 to 1.15).

#### 3.1.4. Personality

With regard to personality, 59/93 (63.4%) of the students had high scores for introversion as compared to 34/93 (36.6%) who were extroverts. On the neuroticism scale 66/93 (71.0%) had a weak degree of neuroticism compared with 27/93 (29.0%) who had strong neuroticism score. Individuals with strong neuroticism traits were 0.37 times more likely to concurrently have strongly introverted scores (OR = 0.37 95%, CI 0.11 to 1.16). Respondents with high introverted scores were 2.42 times more likely to report using a deep approach to learning compared with extroverts. This was significant (OR = 2.42, 95% CI 1.01 to 5.91). There was no significant difference in the odd ratios for: sex (OR = 1.19, 95% CI 0.50 to 2.86), year of study (OR = 0.70, 95% CI 0.30 to 1.63), role in dissection (OR = 1.27, 95% CI 0.87 to 1.87), having seen a dead human body before coming to school (OR = 1.34, 95% CI 0.40 to 4.24), having touched a dead human body before coming to medical school (OR = 0.48, 95% CI 0.18 to 1.24) and being emotionally affected by the dissection work (OR = 0.43, 95% CI 0.17 to 1.04) with respect to the extent of the introverted personality classification of the respondents.

### 3.2. Structural Equation Modelling (SEM)

Those variables marked with a “*” in Table 4 above, were dropped during the quality assessment phase of the SEM, due to identified low or cross loading. Table 5 shows the values for Cronbach alpha and composite reliability for the latent variables that were all less than the maximum threshold of 0.95, a sign of indicator redundancy. Also, the composite reliability values of all the latent variables were above the minimum threshold of 0.6, a measure of the construct’s internal consistency reliability. Also, in Table 5 note that it was only the Average variance extracted (AVE) values corresponding to the latent variables: personality, impact and approach, that passed the minimum threshold of 0.5 for convergent validity. The values of the correlations between the latent variables (see Table 5), were all less than their corresponding latent variable values for the AVE, showing that there was good discriminant validity of constructs represented by the latent variables. This is confirmed by the values in the Heterotrait-montrait ratio of correlation matrix in Table 5 that were all less than 0.9 with the exception of activity and personality. In Table 5, note that those variables whose factor loadings were greater than 0.70 are presented in bold font. Table 6 provides a summary of weights and loadings for each of the above measured variables with respect to their latent variable.

**Figure 2.**
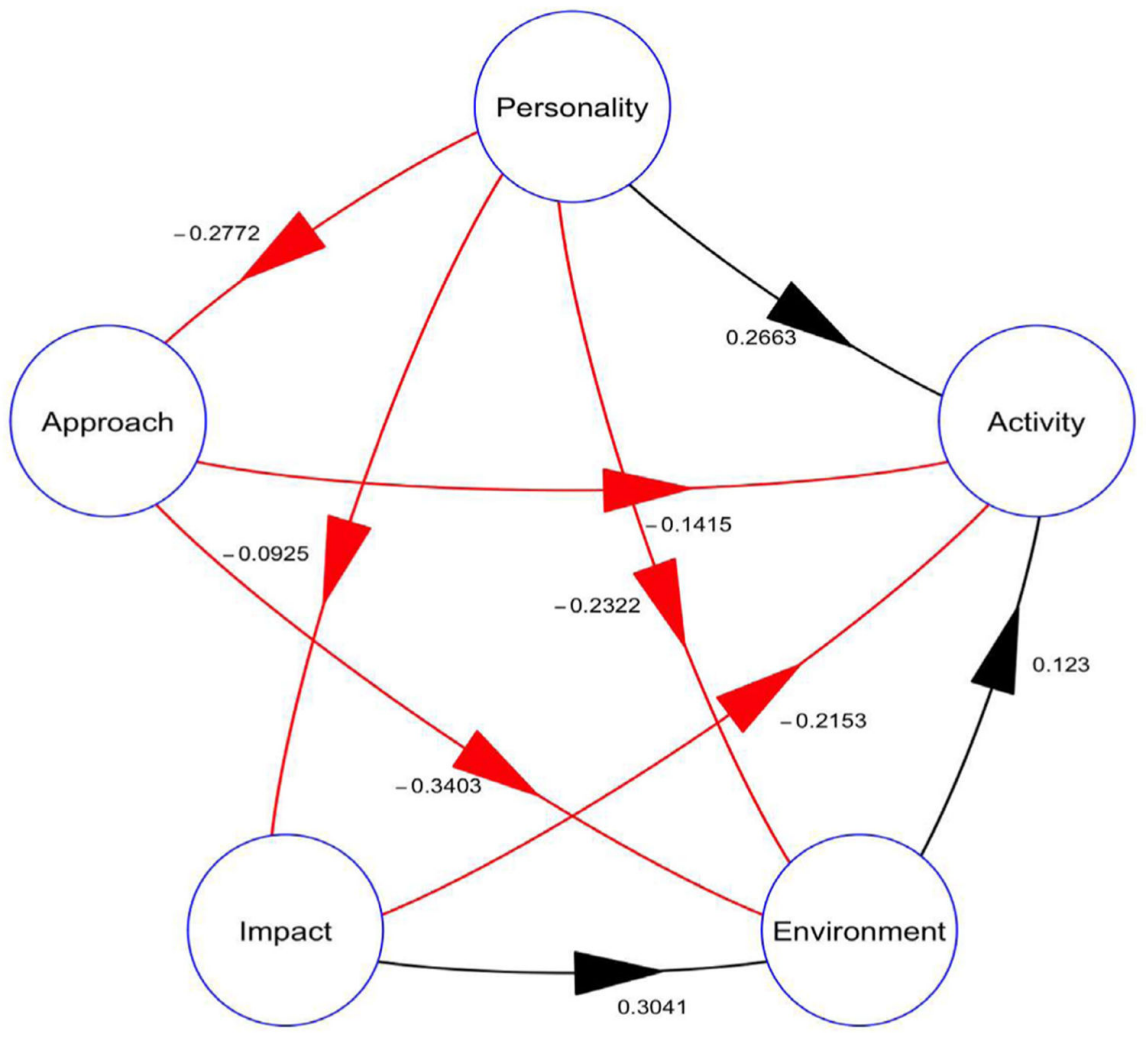
Structural equation model after analysis. Figure summary: This figure 2 provides a visual representation of the final structural equation model in which the various relationships between the constructs measured by the different tools, and their corresponding latent variables (Personality, approach to learning (Approach), impact of the dissection experience (Impact) and perception of the environment (Environment)), that all predict the learner’s activity (Activity), in the dissection room. The red arrows represent negative loadings while the black arrows represent the positive loadings.

Figure 2 provides a pictorial summary of the paths and corresponding coefficients connecting the different latent variables in the structural part of the model. In this figure, personality and perception of the environment that had positive loadings onto activity while approach and impact had negative loadings.

In Table 7, note that it was only the paths from: personality to approach, approach to environment and impact to environment, that remained significant after bootstrapping. It is important to note that because Partial least squares path modelling does not follow any distribution the basis for identifying the significant paths is the absence of zero in the bootstrap 95% CI output. From this table only the paths corresponding to Approach to Environment and that from Personality to Activity are accepted as significant paths.

Table 8 presents a summary of the effects of the hypothesized relationships corresponding to each of the paths between the different latent variables. In this table note that the following three paths representing the hypothesis for: personality having an effect on approach; approach having an effect on reported perception of the learning environment; and impact having an effect on the reported perception of the learning environment had moderately large values for their respective total effects that remained significant after bootstrapping. Similar to the selection of the significant paths in Table 7, here too the acceptance of an effect as significant is based on the absence of the value of zero in the distribution free bootstrap 95% CI Partial least squares path modelling output. The goodness of fit for the final model was 0.25.

## 4. Discussion

We set out to determine the effect of the students’: personality, perception of the learning environment, learning approach and impact of the environment on the student, on undergraduate health professional student’s activity in the human cadaver dissection room. As summarized in Figure 2, we found that the non-neuroticism or introverted personality type and perception of the environment were associated with less hands-on dissection roles, increased reports of being unemotionally affected by the dissection and had touched a dead human body before joining the medical school. Also, a superficial approach to learning and being affected by the dissection room experience (impact), were associated with fewer reports of hands on dissection roles, less reports of being unemotionally affected by the dissection and less likely to have touched a dead human body before joining the medical school. In Figure 2, these two sets of associations are shown by the blue (positive) and red (negative) arrows pointing at the circle for the latent variable for activity. None of these associations (see Table 7) or their effects (see Table 8) was significant.

Previously we have reported that 43% (24/59) of the students simply observed their colleagues dissecting as opposed to doing the preferred hands on dissection [7]. In this study, note that whereas the overall effect for each of the latent variables on activity in the dissection room is not significant, some of the latent variable’s had significant effects on each other (see Table 8). These significant effects present opportunities for interventions leading to enhancement of student hands on activity in the dissection room. For example: 1) the data shows that students with non-neuroticism or introverted personality types were less likely to use surface approaches to learning. According to our data, surface approaches to learning had a negative effect on activity in the dissection room (see Table 8). One solution would be to screen for or better still offer deep study approach training all the students with special emphasis on those students with non-neurotic or introverted personality types. Repeated screening and counselling of students to appreciate the strengths and weakness of their personality as they mature into caring health professionals is already being done at other universities [45]. This when combined with the use of deep learning approaches using curricula innovations like the various student-centered curricula, like problem-based learning, should lead to more hands-on student activity in the dissection room.

The other way to increase student activity based on this model is to modify the student perception of the learning environment. From the model (see Figure 2), the scores for student’s perception of the learning environment can be reduced through: 2) Promoting deep approaches to learning using the previously mentioned student-centered learning approaches like problem base learning. At the time of the study this was already being done through curricula changes to promote deep learning [31] [32]. 3) Offering counselling to students that have been or are negatively impacted by the dissection room environment is another way to improve the student perception of the learning environment. This is important since affected student will tend to avoid hands on activity or not go to the dissection room as a method of coping. Thus, even though the affected students give good scores for the learning environment, their activity is affected as shown in the model (see Figure 2) and from literature [46]. Counselling of affected students as an intervention is important if student hands on activity is to be increased. Each of the above three highlighted pathways represents a modifiable aspect of the learning process that in this setting can have an effect of dissection room activity. Taken together these and other interventions may lead to increased hands on activity and eventually learning through engagement with the learning materials in the dissection room.

Although the model represented in Figure 2 accounts for 25% of the factors affecting student dissection room activity, the information generated by the model is important for teaching of anatomy using cadavers in this and other low resource settings. The use of the study findings is limited by both the presence of missing values in the data and small sample size. The use of multiple imputation to replace the missing values followed by use of partial least squares path modelling with bootstrapping for the small sample size helped mitigate these limitations [26] [44]. The availability of free open source packages and increased computing power made it possible for us to apply partial least squares path modelling techniques [26], that were not as accessible at the time of the study, to our data. Despite these limitations, the work in this study may provide a possible explanation for our previously reported observations [7]. Both the previously reported observations and the findings of the current study have important implications for the teaching of Anatomy in low resource settings. The findings of this study may also explain in part other observations we have made with regards to performance of students on the anatomy examinations [47]. Beyond anatomy, research has shown that, human dissection has an effect on student’s perceptions of themselves [48], and how they later perform on dissection related activities like doing autopsies and handling of terminally ill patients [14] [18] [49] [50].

## 5. Conclusion

This study showed that personality, perception of the learning environment, learning approach and impact of the learning environment on the student, had effects on themselves and on undergraduate health professional student’s activity in the human cadaver dissection room. The modelled effects are opportunities for educational interventions aimed at increasing student activity in the dissection room.

## Figures and Tables

**Table 1.** Demographic characteristics.

Characteristic	Observations
Average Age (years)	21.36 (SD 2.06)
Sex	N (Valid %)
Female	37 (40.22)
Male	55 (59.78)
Missing	4
Year of study	N (Valid %)
Year One	50 (52.08)
Year Two	46 (47.92)

**Table 2.** Summarizing the questionnaires used and study variables.

Questionnaire name (description)	Scales	Study variables from questionnaire
Eysenck Personality Questionnaire (EPQ) personality questionnaire (Personality)	3	Sums that give values to the following three domains: extroversion, introversion and Neuroticism [22]
Revised Study questionnaire (measured learning approaches)	2	Sums that give values for either deep or surface approaches [35]
Impact of Events Scale-revised (IES-R) questionnaire (Measured the effect of the environment on the student)	3	Sums that give values to the following three domains: intrusion, avoidance and hyper arousal [24] [25]
Dundee Ready Education Environment Measure (DREEM) questionnaire (measured respondents’ perception of the learning environment)	5	Sums that give values to the following five domains: perceptions of learning, perceptions of course organizers, academic self-perceptions, perceptions of atmosphere, and social self-perception [23]
Activity and student characteristics	6	Sex, age, year of study, less hands-on role in dissection, first contact with a human body, reported self-awareness

**Table 3.** Summary of observations missing values and comparisons after imputation.

Observations	Missing	GLM Regression (p-value)		ANOVA
		Original	Imputed	Res.dev (df, p-value)
Age	10	0.06 (0.04)	0.11 (0.02)	13.12 (55, 0.04)
Sex	4	0.19 (0.15)	0.25 (0.10)	12.54 (54, 0.10)
Preferred role in dissection	8	−0.04 (0.05)	−0.18 (0.04)	11.86 (53, 0.08)
Saw a cadaver before medical school	2	−0.08 (0.26)	0.02 (0.13)	11.82 (52, 0.64)
Touched a cadaver before medical school	2	−0.12 (0.16)	−0.01 (0.11)	11.53 (51, 0.26)
Emotionally affected by cadaver work	2	0.12 (0.16)	0.04 (0.10)	11.40 (50, 0.45)
Introvert	2	−0.02 (0.05)	−0.01 (0.03)	11.35 (49, 0.62)
Neuroticism	4	−0.05 (0.05)	−0.03 (0.03)	11.18 (48, 0.38)
Deep Strategies	7	−0.02 (0.02)	−0.01 (0.01)	10.71 (47, 0.15)
Superficial Methods	6	0.09 (0.04)	0.05 (0.03)	10.41 (46, 0.24)
Superficial Strategies	10	−0.03 (0.02)	−0.03 (0.01)	09.77 (45, 0.09)
Hyper arousal	2	−0.17 (0.20)	−0.02 (0.14)	09.68 (44, 0.53)
Perception	11	0.01 (0.01)	0.01 (0.01)	09.54 (43, 0.44)
Observations	70	57	92	
Log Likelihood	-	−30.95	−43.61	
AIC	-	89.90	115.22	
McFadden	-	0.57	0.38	
Cox. and Snell	-	0.75	0.43	
Nagelkerke	-	0.82	0.56	

**Table 4.** Summarising the observations from the questionaire.

Variable	N	Mean	Std. dev.	Median	Max	Min
Less hands-on role in dissection	93			4	5	1
Saw a cadaver before medical school	92			2	2	1
Touched a cadaver before medical school*	92			1	2	1
Not emotionally affected by cadaver work	92			1	2	1
I feel same way now as on the first day*	93			1	5	1
Year of study*	93			1	2	1
**Personality scores**						
Introvert	93	12.9	1.6	13	16	9
Neuroticism	93	11.3	1.9	11	15	8
**Study approach scores**						
Deep Methods	93	17.0	3.8	17	25	9
Deep Strategies	93	16.2	3.8	16	25	7
Superficial Methods	93	9.8	3.7	9	21	5
Superficial Strategies	93	22.2	7.0	21	38	10
**Impact of dissection event score**						
Avoidance	93	2.8	0.4	3	3	2
Hyper arousal	93	2.8	0.4	3	3	2
Intrusion*	93	1.2	0.4	1	3	1
**Dundee Ready Education Environment Measure (DREEM) questionnaire**						
Perception of course organizer	93	23.5	4.7	24	33	13
perception of learning	93	32.7	5.4	33	46	10
Social and self-perception	93	15.3	3.8	16	24	5
Perception of academic	93	22.7	3.6	23	31	12
Perception of the atmosphere	93	30.8	5.8	31	44	15

*. These items were dropped at the modeling stage of analysis.

**Table 5.** Showing structural equation modelling quality measures.

Models’ performance					
Latent variables	Personality	Approach	Impact	Environment	Activity
Cronbach alpha	0.55	0.75	0.38	0.72	0.11
Composite reliability (DG. rho)	0.82	0.84	0.76	0.82	0.62
eig. 1st	1.38	2.31	1.23	2.46	1.08
eig. 2nd	0.62	1.11	0.77	1.08	0.99
Redundancy (Q2)	0	0.04	0.01	0.10	0.06
R2	0	0.08	0.01	0.20	0.18
	Correlations between latent variables				
Latent variables	Personality	Approach	Impact	Environment	Activity
Personality	-	−0.28	−0.09	−0.17	−0.31
Approach	−0.28	-	0.15	−0.23	0.28
Impact	−0.09	0.15	-	0.28	−0.23
Environment	−0.17	−0.23	0.28	-	0.05
Activity	−0.31	−0.28	−0.23	0.05	-
AVE	0.66	0.58	0.62	0.48	0.37
Heterotrait-montrait ratio of correlation matrix (HTMT matrix)					
	Personality	Impact	Approach Environment		Activity
Personality	-	-	-	-	-
Impact	−0.15	-	-	-	-
Approach	−0.38	0.02	-	-	-
Environment	−0.13	0.45	−0.40	-	-
Activity	0.90	−1.16	−0.58	0.16	-

**Table 6.** Showing the factor loadings and weights of the measured model.

Measured variables	Weight	loading	Communality	Redundancy
**Personality**				
Introvert	0.32	0.63	0.40	0.00
Non-Neuroticism	0.84	0.96^a^	0.91	0.00
**Study approach**				
None Deep Methods	0.23	0.55	0.31	0.02
None Deep Strategies	0.23	0.63	0.39	0.03
Superficial Strategies	0.42	0.92^a^	0.84	0.06
Superficial Methods	0.39	0.87^a^	0.76	0.06
**Impact of dissection event score**				
Avoidance	0.57	0.73^a^	0.54	0.01
Hyper arousal	0.70	0.83^a^	0.69	0.01
**Environment Dreem tool score**				
Perception of course organizer	0.33	0.62	0.39	0.08
Perception of learning	0.28	0.81^a^	0.66	0.13
Social and self-perception	0.04	0.28	0.08	0.02
Academic self-Perception	0.38	0.87^a^	0.75	0.15
Perception of the atmosphere	0.31	0.74^a^	0.54	0.11
**Activity**				
Less hands-on role in dissection	0.72	0.78^a^	0.61	0.11
Not emotionally affected by dissection	0.63	0.69	0.48	0.08
Touched a cadaver before medical school	0.07	0.12	0.01	0.01

a. These are acceptable factor loadings which are greater than the minimum recommended value of 0.70.

**Table 7.** Showing a summary of the structural models’ regression and boot-strap output.

	Regression results			Bootstrap results (br = 15,000)			Accept
	Estimate	SE	t (p-value)	Mean	SE	95% CI	
**Approach**							
Intercept	<−0.01	0.10	<−0.01 (1.00)				
Personality	−0.28	0.10	−2.72 (0.01)	−0.28	0.11	−0.48 to −0.07	Yes
**Impact**							
Intercept	<−0.01	0.11	<−0.01 (1.00)				
Personality	−0.10	0.11	−0.88 (0.38)	−0.10	0.12	−0.31 to 0.16	No
**Environment**							
Intercept	<−0.01	0.10	<−0.01 (1.00)				
Personality	−0.23	0.10	−2.33 (0.02)	−0.20	0.14	−0.42 to 0.12	No
Approach	−0.34	0.10	−3.39 (<0.01)	−0.34	0.10	−0.52 to −0.11	Yes
Impact	0.30	0.10	3.14 (<0.01)	0.30	0.09	0.11 to 0.47	Yes
**Activity**							
Intercept	<−0.01	0.10	<−0.01 (1.00)				
Personality	−0.26	0.11	2.53 (0.01)	0.24	0.12	−0.06 to 0.44	No
Approach	−0.14	0.11	−1.29 (0.20)	−0.17	0.12	−0.39 to 0.08	No
Impact	−0.22	0.10	−2.06 (0.04)	−0.22	0.10	−0.39 to 0.01	No
Environment	0.12	0.11	1.12 (0.26)	0.11	0.14	−0.17 to 0.35	No

**Table 8.** Summarizing the effects for each hypothesized relationship.

Relationships	Original effects			Bootstrap output of total effects	
	Direct	Indirect	Total	95% CI	Accept
Personality → Approach	−0.28	0.00	−0.28	−0.48 to −0.07	Yes
Personality → Impact	−0.09	0.00	−0.09	−0.31 to 0.16	No
Approach → Impact	0.00	0.00	0.00	0.00 to 0.00	No
Personality → Environment	−0.23	0.07	−0.17	−0.37 to 0.23	No
Approach → Environment	−0.34	0.00	−0.34	−0.52 to −0.11	Yes
Impact → Environment	0.30	0.00	0.30	0.11 to 0.47	Yes
Personality → Activity	0.27	0.04	0.31	−0.03 to 0.49	No
Approach → Activity	−0.14	−0.04	−0.18	−0.42 to 0.04	No
Impact → Activity	−0.22	0.04	−0.18	−0.36 to 0.02	No
Environment → Activity	0.12	0.00	0.12	−0.17 to 0.35	No

## Abbreviation

AIC: Akaike Information Criterion
AVE: Average Variance Extracted
DREEM: Dundee Ready Education Environment
EPQ: Eysenck Personality Questionnaire
HTMT: Heterotrait-montrait
IES-R: Impact of Events Scale-Revised
PBL: Problem Based Learning
PLS-SEM: Partial Least Squares-Structural Equation Modelling
SEM: Structural Equation Modelling
